# Metallothionein and Other Factors Influencing Cadmium-Induced Kidney Dysfunction: Review and Commentary

**DOI:** 10.3390/biom15081083

**Published:** 2025-07-26

**Authors:** Gunnar F. Nordberg, Monica Nordberg

**Affiliations:** 1 Department of Epidemiology and Global Health, Umeå University, SE-90185 Umeå, Sweden; 2 Institute of Environmental Medicine, Karolinska Institutet, SE-17177 Stockholm, Sweden; monica.nordberg@ki.se

**Keywords:** cadmium and kidneys, metallothionein, cadmium transport to kidneys, metallothionein gene expression in lymphocytes, metallothionein autoantibodies, mechanism of cadmium-related kidney effects, zinc–cadmium interaction, arsenic–cadmium interaction, cadmium risk assessment

## Abstract

Cadmium is widely recognized as an important environmental toxicant that may give rise to kidney dysfunction, bone disease, and cancer in humans and animals. Kidney dysfunction occurs at very low exposures and is often considered as the most sensitive or critical effect. Cadmium exposures of concern occur in many countries. In low- and middle-income countries with small-scale mining, excessive exposure to cadmium and other metals occurs in occupational and environmental settings. This is of particular importance in view of the growing demand for metals in global climate change mitigation. Since the 1970s, the present authors have contributed evidence concerning the role of metallothionein and other factors in influencing the toxicokinetics and toxicity of cadmium, particularly as it relates to the development of adverse effects on kidneys in humans and animals. The findings gave a background to the development of biomarkers employed in epidemiological studies, demonstrating the important role of metallothionein in protection against cadmium-induced kidney dysfunction in humans. Studies in cadmium-exposed population groups demonstrated how biomarkers of kidney dysfunction changed during 8 years after drastic lowering of environmental cadmium exposure. Other epidemiological studies showed the impact of a good zinc status in lowering the prevalence of cadmium-related kidney dysfunction. Increased susceptibility to Cd-induced kidney dysfunction was shown in a population with high exposure to inorganic arsenic when compared with a group with low such exposure. Several national and international organizations have used part of the reviewed information, but the metallothionein-related biomarkers and the interaction effects have not been fully considered. We hope that these data sets will also be included and improve risk assessments and preventive measures.

## 1. Introduction

Cadmium (Cd) is a global environmental pollutant. Low-level exposures occur in a considerable proportion of the population in industrialized countries [1,2,3,4]. There are high human exposures among workers in metal industries and in some groups of the general population [3,5]. Excessive exposures to cadmium and other metals occur in occupational and environmental settings of low- and middle-income countries with small-scale mining [6,7]. These problems are of particular concern in view of the increased demand for metals in global climate change mitigation [1]. It is well known that cadmium may give rise to adverse health effects such as kidney dysfunction, bone disease, and cancer in humans and animals [2,3,8]. Kidney dysfunction occurs at very low exposures and is often considered as the most sensitive or critical effect [2,3,8].

Since the 1970s, the present authors have contributed evidence to be presented in the present review concerning the role of metallothionein and other factors (Zn and As) influencing the toxicokinetics and toxicity of cadmium, particularly in relation to kidney dysfunction.

## 2. Role of Metallothioneins in Modifying the Toxicity of Cadmium

Metallothioneins (MTs) are small SH-rich proteins (about 6500 Da) binding Zn, Cd, and Cu in clusters [9,10]. There are four main forms of vertebrate metallothioneins, MT1-4. MT1 and 2 are expressed in most mammalian tissues [10,11,12], MT3 occurs in brain tissue [10,13,14], and MT4 in keratocytes in skin [10,15]. We isolated MT1 and MT2 from animals and used these proteins in toxicological studies. Our previous paper [16] gives more detailed information about the history, chemistry, and methods of isolation. Because of their similar ability to bind cadmium and their similar occurrence in tissues, MT1 and MT2 have similar roles in relation to cadmium toxicology. Most of our studies used a mixture of these two forms, and we, therefore, use the term metallothionein (MT) without specification.

### 2.1. Cd Uptake

In humans, uptake of Cd from food via the oral route is most important. The average proportional uptake is 5 percent in men and 10 percent in females in Western populations. Lower iron stores in females explain the difference based on the interaction of Cd and Fe at the common receptor DMT-1 (reviews [3,17,18]). In young women on a diet with only 50 percent of the recommended intake of iron, up to 47 percent uptake was found [19]. It is well known that Cd induces MT in intestinal mucosal cells. Ohta and Ohba 2020 [20] confirmed the induction of MT-1 and MT-2 in intestinal cells and found the induction of several metal transporters, probably involved in Cd uptake. The exact role of these transporters remains to be clarified. Oral exposure to inorganic Cd in mice resulted in very similar organ distribution regardless of whether wild-type or MT-null mice were used [21], indicating that MT is not of major importance. However, in animal experiments, ingested MT-bound Cd was taken up and distributed to the kidneys to a larger extent than inorganic Cd, indicating that it was partly taken up intact [22]. Binding forms of Cd in food, thus, may be of importance but have not been well investigated. When Cd is airborne, it may be taken up via the lungs. Up to 40 percent of inhaled cadmium may be taken up into the blood (and for nanoparticles, even slightly more, for example, Cd in cigarette smoke). When Cd is taken up in pulmonary tissues, it induces MT that modifies the toxicity of Cd to the lungs [23] (reviews [3,16]). After exposure via the skin, uptake is very limited (review 3).

### 2.2. Cd Transport in Blood, Uptake in Tissues, and Distribution Among Organs

**Figure 1:**
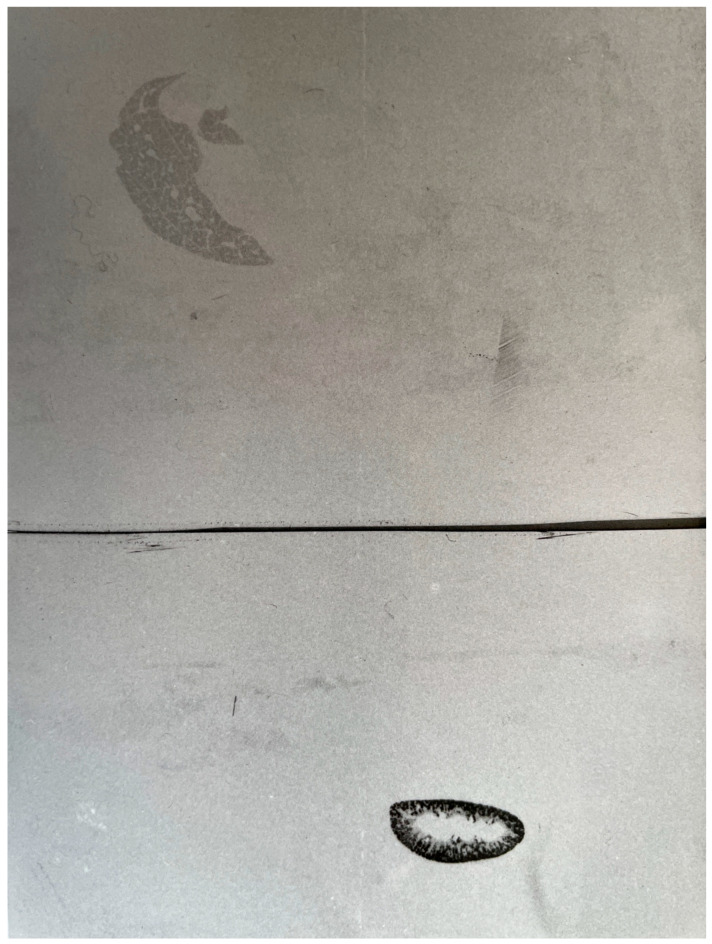
Whole-body autoradiograms of two mice, both at 20 min after injection. Upper: a mouse given i.v. injection of radiolabeled (Cd-109) CdCl_2_. Lower: A mouse given i.v. injection of radiolabeled (Cd-109) metallothionein I and II. Photograph of original whole-body autoradiograms. Experimental details in Nordberg and Nordberg 1975 *Environmental Health Perspectives* [24].

After uptake, cadmium is transported via blood to various tissues where it exerts its toxic effects. In our previous review [16] we republished our chromatographic evidence from in vivo studies in the 1970s. After exposure of mice to inorganic Cd, there is a time-related shift in the distribution of Cd in blood plasma with preferential binding to albumin (Cd-Alb) at 20 min after exposure and the occurrence of Cd-MT at 96 and 192 h. Our autoradiographic studies [24] clearly showed that Cd-Alb is preferentially taken up by the liver and Cd-MT by the kidneys, particularly in the kidney cortex, probably the proximal renal tubules, Figure 1.

Figure 1 (upper) illustrates that when cadmium chloride is injected i.v., a dominating amount of the cadmium is taken up in the liver, and only the liver can be seen in this whole-body autoradiogram. We showed in our previous review [16] and earlier [25] that at 20 min after injection, cadmium is bound to albumin in blood plasma. This picture (Figure 1, upper) thus shows the distribution of albumin–cadmium to the liver. The lower part shows the distribution of cadmium bound to metallothionein. In this whole-body autoradiogram (lower), only the kidney can be seen. This part of the autoradiogram shows the specific uptake of cadmium in the kidney, with the highest concentration being in the cortex, where the proximal tubules are. The evidence, upper and lower pictures, speaks in favor of MT as a much more efficient carrier of Cd to the kidney than albumin.

Johnson and Foulkes, 1980 [26], performed long-term infusion in animals of low doses of Cd-MT, only 60 ng/min, leading to an increase in plasma Cd-MT of approximately 0.1 nmol/L, an increase in plasma Cd of only 0.07 ng/mL, i.e., total plasma Cd within the physiological range. They showed that Cd was taken up in the kidneys. This is also in line with general knowledge about the glomerular filtration of small proteins in plasma and their uptake from the filtrate into proximal tubules in the kidney cortex. It is unfortunate that Fels et al. [27] did not consider the data by Johnson and Foulkes [26]. In their discussion based on their literature search, Fels et al. [27] stated the following: “renal uptake of ultrafiltrated Cd2+-MT was only observed with very high concentrations of parenterally applied acute Cd2+-MT injections varying between 0.7–2 µmol/L (citing several references in their paper, but not Johnson and Foulkes [26])”. They used this incomplete information when discussing their in vitro data in a rat proximal tubule cell line. The reason why Fels et al. [27] did not find uptake of Cd-MT in their proximal tubular kidney cells in vitro at low concentrations of CdMT is not obvious but may be related to differences between Johnson and Foulkes [26] and Fels et al. [27] in experimental models (kidney in vivo versus proximal tubular cell line) and methodology used in these studies. A more recent publication from a related group of scientists [28] seems to accept that CdMT is transported in plasma, filtered in the glomeruli, and taken up by kidney tubules also at low plasma Cd-MT concentrations.

Fels et al. [27] suggested that Cd uptake in the kidney tubules is instead mediated by Cd-Alb, Cd-β_2_-Microglobulin, and Cd-lipocalin-2. Concerning Cd-Alb, the studies by Nordberg and Nordberg 1975 [24] and Figure 1 show that Cd-Alb is efficiently taken up in the liver and only a small proportion is filtered through the glomeruli and taken up in the kidney tubules. Cd-MT is taken up very efficiently by the kidney tubules (Figure 1 and [26]. We have not studied Cd-β2-microglubulin and Cd-lipocalin-2 and cannot contribute to a discussion of a possible role of these proteins, but they can only be filtered through the glomeruli if they occur in plasma, and this remains to be demonstrated. We would like to emphasize the important role of Cd MT at times exceeding 4 days after a single injection and in long-term exposure to Cd. A transport role for MT carrying Cd to the kidney is also supported by observations by Liu et al. in MT-null mice [29]. These authors found that after a single injection of CdCl2, Cd concentrations in the kidneys continue to increase with time in control but not in MT-null mice, indicating that an important source of Cd in the kidney is the uptake of Cd-MT. There are many other publications reporting increased kidney concentrations of cadmium after a single injection of ionic cadmium. For example, Gunn and Gould [30] found a more than four-fold increase in the concentration of cadmium in the kidney cortex of rats between 1 and 150 days after injection. The findings by Liu et al. [29] in control (wild type) mice fit well with our own observations on the pattern of cadmium binding in plasma after a single injection of CdCl2 in such mice (see Figure 1 in [16]). Initially, after injection, Cd in plasma is bound to proteins of the size of albumin, and such cadmium is mainly taken up by the liver, but a small proportion goes to the kidneys. This early distribution phase is expected to be the same in wild-type and MT-null mice. After 4 days, a considerable proportion of Cd in plasma is bound to a protein of the size of MT, and in that period (after 3–4 days), there is more uptake of Cd in the kidneys of the wild-type mice than in the MT-null mice (which have no MT). In long-term exposures, this pattern is repeated multiple times and explains the higher concentrations of cadmium in the kidney cortex than in the liver in normal (wild-type) animals. Further studies by Liu et al. 1998 [31] on chronic Cd exposure of MT-null and control mice show that with the same exposure, MT-null mice accumulate much less Cd in kidneys (10 µg/g) than wild-type mice (140 µg/g). These findings are also in line with the transport of Cd to the kidney by Cd-MT in wild-type mice but lacking in MT-null mice.

A role for MT in carrying Cd to kidney tubules is supported by information (in vivo) in addition to what was cited previously in this paper [32,33,34,35,36] and occurs even at very low plasma concentrations of CdMT [26]. Such a course of events seems to be widely accepted (review [37]). In their comprehensive review, Thevenod and Wolff [38] discussed a specific molecular mechanism for uptake in the proximal renal tubules and the limited uptake in vitro by this mechanism at low Cd-MT concentrations in the exposure medium. However, they noted that only 0.02–0.3 percent of filtered proteins, including MT, are excreted with the urine [39], and they state that additional pathways for proteins and Cd-protein complexes must exist. Thus, Thevenod and Wolff [38] recognize that Cd-MT is taken up by kidney tubules even at low plasma concentrations. Also, a recent publication by Li et al. 2025 [40] mentions transport of Cd-MT to the kidneys. On the other hand, a paper by Satarug et al. [41] considered a possible role for albumin and other proteins according to the suggestions by Fels et al. [27]. Although there is no direct chromatographic data on Cd-MT in human blood plasma, it is likely that MT has a transport role for Cd also in humans (the uncertainties in the data by Li et al. 2020 [42] were pointed out previously [16]). The existence of MT in the plasma of humans and the fact that there are increased levels of MT in the blood plasma and urine of cadmium-exposed humans [43,44,45] support a transport role. The finding that cadmium in the urine of humans is bound to MT [46,47,48] gives further support. See also Section 3.2 about MT as an indicator of tubular kidney dysfunction. The fact that renal tubular dysfunction is related to an increased level of MT in urine may partly be explained by a lower uptake of CdMT from tubular fluid by dysfunctional kidney tubules compared to fully functional tubules—see further discussion in Section 3.2. The previously mentioned study in animals [22] showing that ingested Cd-MT is distributed to the kidneys to a larger extent than ingested ionic Cd also supports a transport role for MT to the kidneys. In summary, available data supports an important role of Cd MT for the transport of Cd to kidney tubules at times exceeding 4 days after a single exposure and in long-term exposure to Cd. Available data thus makes it more likely that MT is a major carrier of Cd to the kidney tubules than albumin and other proteins.

Based on presently available evidence, we consider that our explanatory model is still valid as it was first advanced in 1984 [49] and reproduced in our paper 2022 [16]. It includes the initial transport of Cd from the site of absorption to the liver by Cd bound to Alb. In the liver, Cd is split from Alb and bound to newly synthesized MT, which is partly released to blood plasma and transported to the kidneys bound to MT.

### 2.3. Metallothionein and Kidney Toxicity of Cadmium

Our early studies in the 1970s showed the different toxicity to the kidney of injected Cd-MT compared to ionic Cd. Cd-MT causes an immediate uptake of Cd in kidney tubules, leading to toxicity, while ionic Cd is taken up mostly in the liver and does not give rise to toxicity at the same doses [50]. Studies by other scientists further described the uptake of Cd-MT in kidney tubules and the renal tubular lesions after injection of Cd-MT in animals [51,52,53,54]. After pretreatment with repeated small doses of ionic Cd, synthesis of MT in kidney proximal tubules is induced, and a high level of tissue MT is established, protecting sensitive membrane binding sites from the toxicity of a bolus dose of injected Cd-MT [55]. A protective role of MT-synthesis against kidney toxicity from cadmium was also shown in the studies by Liu et al. [31,56], where MT-null mice accumulated less Cd in the kidneys but suffered toxicity at lower doses of Cd than wild-type mice. The lack of MT gave rise to less Cd in the kidneys because it could not be transported there by MT. Cadmium in the kidney was not protected by MT, and therefore, toxicity occurred at lower levels of Cd in the kidneys. A long-term exposure experiment in animals with intact MT-synthesis by Elinder et al. 1987 [57] (see also [16]) shows the importance of the Cd/Zn quotient in MT isolated from kidneys of Cd-exposed animals for the appearance of toxicity. In this experiment, the cadmium concentration in the kidney cortex and the Cd/Zn quotient in MT gradually increased with continuous exposure to Cd. When the Cd/Zn quotient was low, the MT had sufficient Zn sites to pick up incoming Cd (replacing Zn), and the cells were protected. When the cadmium concentration in the kidney cortex reached a level where the molar quotient of Cd/Zn was 6, protection by MT was insufficient, and toxic effects in kidney tubules appeared because Cd binds to sensitive sites in cellular membranes and causes toxicity. This is the critical concentration. The toxicity of Cd to the kidney tubules in long-term exposures is, thus, not directly caused by the influx of CdMT and its degradation, but is due to the total load of Cd in the cells and the lack of sufficient protection by MT because Cd has replaced Zn in the protein.

The cited findings support the model of cadmium transport and toxicity referred to above [49] implying that in long term exposure, Cd-MT is carried from the liver by MT to the kidney tubules, degraded in lysosomes [58], releasing Cd that is picked up by previously synthesized MT (with Zn that can be replaced by Cd). When the critical concentration of cadmium is reached and binding sites for Cd on MT are exhausted, Cd binds to sensitive membrane sites and causes toxicity. These considerations are valid even if they do not consider all the recent advances in molecular pathways (reviews [37,38,59]). The protective effect of MT by binding Cd in tissues explains why Cd accumulates in tissues, but there are no adverse effects on tissues until Zn sites in MT have been exhausted.

The binding of Cd to MT further explains the retention and the long biological half-life (10–40 years in humans) of Cd in the kidney [3,60].

## 3. Metallothionein-Related Biomarkers in Epidemiological Studies

In collaboration with scientists at the World Health Organization and the European Commission INCO-DC program, we established research together with scientists in China. A first set of publications showing dose–response relationships between biomarkers of exposure and effects (Cd-induced kidney dysfunction) was based on data that had been collected in Dayu County, Jiangxi province, in 1986/1987 [61,62,63]. Studies performed in other cadmium-polluted areas of China since the 1990s included assessments of renal dysfunction and other adverse health effects and showed dose–response relationships between cadmium exposure measured as urine-Cd (UCd), blood-Cd (BCd), and biomarkers of kidney dysfunction such as increased urinary excretion of albumin (Alb), β_2_-Microglobulin (B2M), or N-acetyl-β-D-glucosaminidase (NAG). A set of findings was reported at a symposium in Shanghai, China, and published in 2004 [64,65,66,67,68]. The first studies of MT gene expression in peripheral lymphocytes as a biomarker of susceptibility to cadmium toxicity [69,70] were reported during this symposium.

### 3.1. Metallothionein-Related Biomarkers of Susceptibility to Cd Toxicity

As described in previous sections of this paper, MT is induced by cadmium in many tissues in the body when there is exposure to cadmium. MT is an efficient scavenger of Cd and protects tissues against the toxicity of ionic Cd. It is well known [3] that most of the cadmium in blood is found in blood cells. In blood lymphocytes, Cd is bound mainly to MT [71]. The synthesis of MT and MT mRNA in lymphocytes can be induced by cadmium [72]. This information was the basis for the studies by Lu et al. 2001, 2004, 2005 [69,70,73] who examined whether gene expression of MT in peripheral blood lymphocytes (PBLs) would be a suitable indicator of MT in other tissues like the kidney. MT mRNA, i.e., gene expression of MT, was measured by RT PCR in PBLs before (basal) and after in vitro cadmium exposure of the PBLs (induced). As expected, MT mRNA, both basal and induced values, were related to BCd and UCd among Cd-exposed workers [69], because in these workers, Cd exposure efficiently induced MT in tissues. Another study in workers also showed a relationship between MT mRNA in PBLs and occupational cadmium exposure [74]. In a study of cadmium-exposed workers in China, Lu et al. [69] showed that at similar UCd values, workers with high levels of induced MT mRNA in PBLs had lower values of urinary NAG and thus were protected from this adverse effect of Cd exposure. This finding indicates that gene expression of MT in PBLs can be used as an indicator of general tissue susceptibility to cadmium toxicity. The findings were similar when these methods were used to examine the same possible relationships in a cadmium-exposed group of the general population in China [73]. In this study, an inverse relationship was observed between induced MT mRNA in PBLs and urinary NAG. The findings further confirm the usefulness of these measurements in indicating general MT protection in body tissues.

Another metallothionein-related biomarker that can indicate susceptibility to the development of Cd-induced tubular kidney dysfunction is autoantibodies against metallothionein in blood plasma. In a group of 262 type-2 diabetic patients in China, an increase in MT-antibodies in plasma from low to high, increased the Odds Ratio for kidney tubular dysfunction to 5.5 (CI 2.25–13.73); for comparison, an increase in the level of urinary Cd from <1 to >1 microg/g creatinine increased the OR to 3.34 (CI 1.17–9.53) in the same study [75].

### 3.2. Urinary Metallothionein as Biomarker in Epidemiological Studies

As described in Section 2.2, Cd-MT is efficiently filtered in the glomeruli and reabsorbed in the proximal tubules of the kidney in the same way as other small proteins. Increased excretion of MT in urine is, therefore, expected to occur when there is damage to the kidney tubules. Shaikh and Tohyama [76] pointed out the usefulness of urinary MT as an indicator of cadmium body burden and of cadmium-induced nephrotoxicity. Cd-related damage of renal tubules occurs when the Cd concentration in the kidney cortex is accumulated to 150–200 µg/g. At that level, urinary Cd and urinary MT show a strong increase (review [77]). Urinary MT is also an index of Cd exposure, because at exposure levels below those giving rise to renal tubular dysfunction, there is also a relationship between urinary Cd and urinary MT [78]. Increased levels of MT in urine were reported in Cd-exposed groups of Cd workers and population groups residing in Cd-polluted areas [43,46,48,76,78] as well as in patients with type-2-diabetes with tubular kidney dysfunction [75]. Such increased levels of MT in urine may be explained by a lower uptake of Cd-MT from tubular fluid by dysfunctional kidney tubules compared to fully functional tubules and/or by a release into urine of Cd-MT from dysfunctional tubules. Both mechanisms agree with the use of urinary MT excretion as a useful indicator of Cd-induced tubular kidney dysfunction, like other small proteins like β_2_-Microglobulin.

Recently it was shown that urinary MT can be used as an indicator related to mortality. A relationship to all-cause mortality and mortality in renal and urinary tract diseases was found in a Cd-contaminated area of Japan [40].

## 4. Other Factors Influencing Cadmium-Induced Kidney Dysfunction

### 4.1. Decreased Exposure

It is likely that decreased Cd exposure will influence the development of Cd-induced kidney dysfunction, but it is not well documented what changes there will be in exposure and effect indicators. The following study was therefore conducted.

Populations in a highly cadmium-polluted area, a moderately polluted area, and a non-polluted area of Zhejiang province, China, were studied in 1995 [79]. In all three areas, rice is a major component of the diet. We measured Cd in rice and found an average level of 3.7 mg/kg in the highly polluted area, 0.51 mg/kg in the moderately polluted area, and 0.072 mg/kg in the non-polluted area. Increased levels of urinary cadmium were found with 10.7 µg/L in the highly polluted area. Urinary B2M and Alb were statistically significantly increased among the residents of the polluted areas.

We performed a somewhat extended study with 790 participants in the same areas in 1998 [80] at the time when consumption of rice with high cadmium had been stopped (1995–1996 in the highly polluted area and around 1998 in the moderately polluted area). In 2006 (8 years later), some of the 790 participants in the previous study had died, and others had moved to other areas in China. There were 412 people who were available for follow-up in the three areas, and they joined our study that year [81]. The data collected showed changes in blood and urine cadmium as well as biomarkers of kidney dysfunction compared with the same persons in 1998, as shown in Table 1.

During the observation period, BCd values decreased considerably in the polluted areas, and to a lesser extent in the non-polluted area. This is expected, since BCd is an established indicator of recent Cd exposure [3] and because intake from contaminated rice was stopped. UCd decreased to some extent in residents of the highly polluted area, but not in the moderately polluted area. This finding is in line with the fact that UCd is an indicator of Cd accumulation in the kidney and that the half-life of Cd in the kidney is very long (10–40 years [3]). When there is Cd-induced dysfunction of the kidney tubules, the half-life in the kidney is shortened, more so with more pronounced dysfunction. The absence of a decrease in UCd in residents of the moderately polluted area is explained by the longer half-life in the kidney and a low uptake of Cd from background levels in food, including the so-called non-contaminated rice.

There were increased levels of NAG and B2M in both polluted areas despite the decreased exposure and decreased levels of BCd in both areas and UCd in the highly polluted area. NAG and B2M are indicators of tubular kidney dysfunction. Experience from occupational cadmium exposures indicates that such dysfunction is irreversible [82]. The increased levels of these indicators of tubular dysfunction may also be related to the fact that tubular function decreases with age, with some limited increase in the biomarkers of tubular dysfunction. The influence of age was shown in a 19 year follow up of participants in another study of Cd pollution in China: B2M increased from 0.37 to 1.35 mg/g creatinine in the control area and from 1.35 to 6.53 mg/g creatinine under continuous exposure during 19 years in the polluted area (Dayu County, Jiangxi province) [83]. It should also be remembered that the background exposure to Cd in non-polluted areas in southern China is higher than in Western countries like Sweden.

The albumin excretion in residents of the highly polluted area (Zhejiang province) was 8.95 mg/g creatinine (Geom. mean) in 1995 [79], 5.95 mg/g creatinine among all the 294 participants in this group in the extended study 1998 [80], and 5.38 among the 190 participants who were followed up later [81], Table 1, i.e., some participants with higher values were lost to follow up. A decrease to 3.22 mg/g creatinine was observed in 2006. This decrease is statistically significant compared to 5.38 in 1998 for the same individuals. A similar tendency of a decrease in urinary Alb is seen in the moderately polluted area; however, it is not statistically significant (Table 1). These observations show that the effect of Cd exposure on albumin excretion is reversible when exposure decreases. The contrast in development compared to the tubular indicators B2M and NAG is obvious.

Because of the difference in development after a decrease in exposure between urinary Alb and the indicators of tubular dysfunction (B2M and NAG), it is unlikely that the two effects are explained by the same mechanism. It is well established that Cd-induced tubular kidney dysfunction is dependent on the accumulation of Cd in tubular cells, leading to impaired reabsorption of B2M in the tubules and increased urinary excretion (review [3]), and as mentioned, the tubular damage seems to be irreversible. The increased urinary albumin excretion induced by cadmium exposure is considered mainly to be an effect on the glomerular capillary wall [81,84,85] and a parallel loss of red blood cell charge by a decrease in sialic acid in blood cells and the glomerular membrane [86]. This is the probable explanation why the albuminuria is related to blood-Cd and not to urine-Cd. Thus, it is reversible when BCd decreases. Hotz et al. [87] also reported a decrease in urinary Alb after decreased Cd exposure.

Other ideas concerning the mechanism for Cd-induced albuminuria were recently advanced by Satarug et al. 2024 [41], who performed mathematical modeling on their data from Cd-exposed populations in Thailand and concluded that Cd in renal tissue affected tubular reabsorption of albumin and β2M similarly. They suggest that there is Cd-reduced receptor-mediated endocytosis and subsequent lysosomal degradation of each protein by a shared mechanism. Because these authors did not have an interval of decreased exposure in their data set, they were unable to observe the different development of Cd-induced B2M-uria and Alb-uria after such a decrease in Cd exposure. In our data, shown in Table 1, this difference is obvious and makes it highly unlikely that the two phenomena are explained by the same mechanism. Plausible mechanisms are discussed above.

### 4.2. Zinc Status

Zinc (Zn) and cadmium have chemical similarities, and the evidence supporting the fact that the toxicity of Cd is related to interference with the essential functions of Zn in proteins/enzymes and transcription factors was reviewed in [59]. Animal experiments showing Cd/Zn interactions are reviewed elsewhere and show that Cd-related tubular kidney dysfunction is decreased when there is combined exposure to Cd and Zn [88]. Recent evidence in vitro and in Cd-induced murine nephrotoxicity [89] indicates a role of ferroptosis as a mechanism of cellular damage that is alleviated by treatment with Zn-protoporphyrin IX. In Section 2.3 and in our previous review [16], we described the relationship between Cd/Zn quotient in MT in the kidneys and toxicity to kidney tubules in animals exposed to Cd for a long time. It is likely that zinc status would influence the prevalence of Cd-induced kidney dysfunction also in humans, but we found very few published papers presenting such evidence, and the studies to be summarized in the following text are, therefore, of interest.

Serum zinc (SZn) and hair zinc (HZn) had been measured in the studies in Dayu, China, 1987, mentioned in Section 3, but not used for statistical evaluation. Studies together with our colleagues in China [90] used these data in statistical analyses to examine a possible influence of zinc status. Comparisons between the Cd-polluted area and the control area found statistically significantly higher BCd, UCd, and urinary B2M excretion (all *p* < 0.01) in the polluted area. Serum zinc (SZn) was lower (*p* < 0.01) in the polluted area compared to the control area. Odds ratios for kidney dysfunction were calculated by a multivariate adjusted regression model. Results are shown in Table 2.

The model adjusted for age, gender and smoking. Kidney dysfunction was defined as urinary B2M > 300 μg/g creatinine. The Zn/Cd quotient was formed by dividing SZn by BCd. Data on UCd, BCd, and Zn/Cd were all categorized in quartiles. SZn and HZn were categorized by interquartile distribution (<25th percentile, 25–75 percentile, and >75th percentile).

As seen in Table 2, there are statistically significantly increased ORs for kidney dysfunction in the higher quartiles of UCd and BCd. For SZn, lower OR was seen with higher SZn (not statistically significant). For HZn, there was a statistically significantly lower OR in the highest quartile. For Zn/Cd, statistically significantly lower ORs for kidney dysfunction were seen in all three quartiles exceeding the reference quartile. These observations in humans indicate that zinc status influences the development of Cd-induced tubular kidney dysfunction. The findings are in line with findings in animal experiments. There seems to be no other published papers showing such effects on kidney tubules in humans. However, an effect of Zn in improving Cd-related decreased glomerular filtration in kidneys was reported [91]. Another study [92] reported that plasma selenium and serum zinc influenced Cd-related kidney disease, and a review [59] suggested a relationship between Cd and several diseases and low zinc intakes and other diseases, but included few documented interactions between Cd and Zn for a specific disease. A generally increased susceptibility to Cd effects would be expected in zinc deficiency. Since 30 percent of the world population suffers from zinc deficiency according to an evaluation by WHO [93], and there is a particularly high prevalence in some low-income countries, e.g., 72 percent in Ethiopia [94], the combination of Cd exposure and zinc deficiency may be an important problem in a global perspective.

### 4.3. Arsenic

Another factor that influences the occurrence of Cd-induced kidney dysfunction in human populations is exposure to inorganic arsenic. Human populations are exposed not only to varying levels of cadmium but also to varying levels of arsenic depending on drinking water source, proximity to point sources such as non-ferrous smelters, domestic habits, and fish consumption [8,95,96]. Such arsenic exposures may give rise to adverse health effects on the nervous and cardiovascular systems, kidneys, skin lesions, and cancer [8]. How arsenic exposures influence the occurrence of adverse health effects of cadmium is of considerable interest but has not been extensively studied. Inhalation exposure to inorganic arsenic occurs in occupational settings, and sometimes, as in the case to be presented, in homes. Intake via drinking water from tube wells drilled in arsenic-containing bedrock is also common (e.g., in Bangladesh) [95,97,98]. Populations with a high intake of fish and shellfish take up arsenic in a non-toxic form (arsenobetaine) readily excreted in urine.

**Figure 2:**
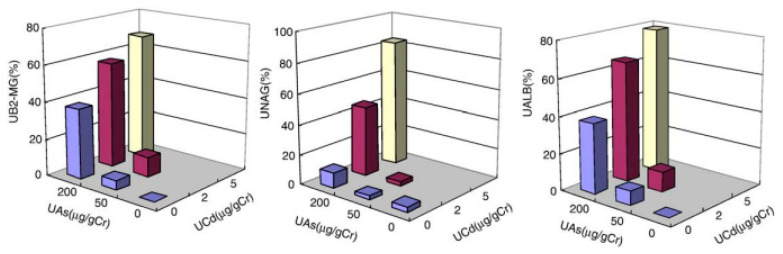
Prevalence (%) of elevated excretion of three biomarker proteins among residents in a metal-contaminated area of China in relation to combined exposure to Cd and As, as indicated by urinary As and Cd excretion. UALB, urinary albumin >15 mg/gcreatinine; UAs, urinary arsenic; UB2-MG, urinary beta-2-microglobulin >0.3 mg/gcreatinine; UCd, urinary cadmium; UNAG, urinary N-acetylglucosaminidase >23 U/gcreatinine. Reproduced from Nordberg G.F., Jin, T., Hong, F. et al. *Toxicol. Appl. Pharmacol*. 2005 [100] with permission from Elsevier Rightslink.

In studies to be summarized here, of metal and metalloid-exposed population groups, we collaborated with Dr Hong et al. [99,100] when investigating people in an area in Guizhou Province, China. In this area, exposure to arsenic and cadmium occurred because of indoor burning of coal with a high content of arsenic and a lower amount of cadmium. Another population group without excessive exposure was studied for comparison. Biomarkers of exposure, such as urinary arsenic (UAs), urinary Cd (UCd), and biomarkers of kidney dysfunction, i.e., urinary B2M, NAG, and Alb, all showed elevated values among residents in the exposed area compared to the non-exposed area. When displaying the results in a three-dimensional diagram, Figure 2, an interaction is seen with more pronounced increases among those with the highest combined exposures to both As and Cd compared to those with only arsenic exposure. This finding is obvious even if there was a lack of observations of subgroups with intermediate Cd exposure and low arsenic.

Urinary arsenic values in this study represented inorganic arsenic because Guizhou Province is an inland Province in China where the consumption of fish and shellfish is very low. Studies in Zhejiang Province [101], also presented in [100], found no dose–response relationship between total UAs and biomarkers of kidney dysfunction. The reason was probably the high consumption of fish and shellfish containing “fish-arsenic” in this coastal area. When specific determinations of inorganic arsenic in urine were performed [101], a relationship was found between inorganic arsenic in urine and increased values of the biomarkers of kidney dysfunction. This confirmed that in the coastal area of Zhejiang Province, the high arsenic values in urine were mostly explained by non-toxic fish-derived arsenic such as arsenobetaine. The interaction between inorganic arsenic and Cd was also found in these studies.

Our findings fit well with animal data by Liu et al. [102] showing that exposure to inorganic arsenic potentiates the nephrotoxicity of cadmium with a more than additive effect. It is interesting that these animal studies showed that the effects were more pronounced in genetically modified animals lacking the ability to synthesize metallothionein (MT null animals). Epidemiological studies based on the NHANES database in the US reported a statistical relationship between combined urinary excretion of arsenic, cadmium, lead, and mercury and blood urea nitrogen in adolescents [103]. Recent studies [104] in several areas in China found associations between As, Cd, and other metals and metalloids in relation to increased urinary NAG. The authors stated that interactions may affect the associations, but presented limited specific data to show such interactions.

## 5. Data Used by National and International Organizations

The data reviewed in the present paper has, to a considerable extent, been used by National and International Organizations as a basis for risk assessments aiming at the limitation of human cadmium exposures in occupational and general environments. An international group of experts appointed by the International Programme on Chemical Safety (IPCS), an organ sponsored by the United Nations Environment Programme, the International Labour Organization, and the World Health Organization, published an Environmental Health Criteria document on cadmium in 1992 [105]. This document described the role of MT in the transport of Cd from the liver to the kidneys and estimated Cd intakes that would cause kidney damage based on toxicokinetics and an estimated critical concentration of Cd in the kidney cortex. Such estimates were very useful at that time, because only a few epidemiological studies had been published. Much of the data before 1992 that is included in the present review was used.

A report was published in 1998 by a group of experts appointed by the Swedish Chemicals Inspectorate [82]. This group based their discussions on the same explanatory model of cadmium kinetics and toxicity discussed in the present paper (Section 2.3). They considered epidemiological evidence and estimated the lowest critical concentration in the kidney cortex related to tubular kidney dysfunction and risks at various intake levels.

The explanatory model [49], summarized in Section 2.3. was developed by us in parallel with a multicompartment toxicokinetic model [106], which was used for calculations of relationships between Cd intakes and concentrations in the kidney cortex. Choudhury et al., 2001 [107], applied new computational tools to the model, and Diamond et al., 2003 [108], used these tools to calculate relationships between Cd accumulation in the US population and intake levels. Calculations with these tools were also used in the risk assessment for cadmium exposures in the US by ATSDR, 2012 [109]. The International Union of Pure and Applied Chemistry, through its Task Group on Risk Assessment of Effects of Cadmium on Human Health [2], used calculations by these models for comparison with dose–response relationships observed in epidemiological investigations. The results compared favorably with each other and formed a good basis for the conclusions by the Task Group.

## 6. Concluding Remarks

This review summarizes scientific contributions by the authors from the 1970s up to the present, particularly relating to factors of importance for the development of Cd-induced kidney dysfunction, an effect of cadmium exposure that occurs at very low exposures. The small, cysteine-rich protein metallothionein (MT) was identified as of key importance, both in transporting Cd from blood plasma to kidney tubules and in binding and detoxifying Cd in kidney tubule cells and other tissues. MT-gene expression in blood lymphocytes was shown to be related to the occurrence of kidney dysfunction in Cd-exposed workers: those with high levels of MT-gene expression had less kidney dysfunction than those with low levels, and this test can be used as a general indicator of tissue protection by MT. Another indicator inversely related to tissue protection by MT is antibodies against MT in blood plasma. High levels increased the occurrence of Cd-related kidney dysfunction. Since these biomarkers make it possible to measure MT protective function in tissues, we hope they will be further used in the future. In a population with a high long-term intake of Cd, the levels of blood-Cd and UAlb decreased after an interval of 8 years with low Cd intake. In contrast, the indicators of tubular damage increased. These findings indicate that there are different mechanisms behind the respective effects. Other studies showed a protective effect of good zinc status against Cd-induced kidney dysfunction. An increased general susceptibility to the development of adverse health effects of Cd exposures would be expected in zinc deficiency. Since 30 percent of the world population suffers from zinc deficiency, particularly in some low-income countries, the combination of Cd exposure and zinc deficiency may be an important problem from a global perspective. Concomitant exposure to Cd and inorganic arsenic gave rise to a more than additive effect on tubular kidney dysfunction, and population groups with such exposures need special attention.

Many of the findings summarized in the present review have been used from the 1990s up to the present by several national and international groups of scientists as a basis for risk assessments for cadmium. However, the MT-related biomarkers and the interaction effects have not been fully considered. We hope this will be carried out in the future to further protect public health. Despite the mentioned international efforts, excessive exposures to Cd and other metals still occur in several low- and middle-income countries with small-scale mining. In view of the increased global demand for metals in climate change mitigation, preventive measures are urgent.

## Figures and Tables

**Table 1:** Measured values of exposure indicators: blood-Cd (BCd), urine-Cd (UCd), and indicators of kidney dysfunction: urinary β_2_-Microglobulin, NAG, and albumin in 1998 and 2006 among residents in the three study areas with varying degrees of cadmium pollution (based on Liang et al. 2012, *Environmental Health Perspectives* [81]).

Characteristic/Indicator	Nonpolluted		Moderately Polluted		Highly Polluted	
	1998	2006	1998	2006	1998	2006
n (% male)	91 (36.8)		131 (33.6)		190 (33.0)	
Median age (years) in 1998	53.0		46		46.5	
BCd (µg/L)	1.31 (0.75–2.19)	0.87 (0.57–1.46) *	3.78 (2.50–6.50)	1.80 (1.27–2.75) *	8.90 (4.97–13.6)	3.31 (2.25–5.15) *
UCd (µg/g creatinine)	1.79 (1.07–3.63)	2.31 (1.42–3.84)	3.62 (2.52–6.05)	3.79 (2.66–6.13)	11.6 (7.61–18.7)	8.97 (5.87–13.1) *
NAG (U/g creatinine)	1.80 (0.89–3.99)	7.92 (5.65–10.7) *	4.12 (2.02–11.3)	8.15 (5.77–10.7) *	7.64 (4.44–14.2)	11.8 (7.25–17.8) *
β_2_-Microglobulin (mg/g creatinine)	0.12 (0.07–0.24)	0.16 (0.09–0.26)	0.16 (0.10–0.31)	0.28 (0.17–0.44) *	0.28 (0.14–0.50)	0.42 (0.20–0.79) *
Albumin (mg/g creatinine)	3.08 (1.40–6.40)	2.84 (1.13–4.71) *	4.47 (2.10–9.30)	3.83 (1.33–9.44)	5.38 (2.48–11.9)	3.22 (1.28–7.22) *

Values for BCd, UCd, NAG, β_2_-microglobulin, and albumin are reported as GM (IQR). * *p* < 0.05 compared with 1998, by the Wilcoxon signed-rank test.

**Table 2:** Measured values of bioindicators of exposure (BCd: blood cadmium and UCd: urinary cadmium); indicators of zinc status serumZn (SZn), hairZn (HZn), and Zn/Cd quotient (SZn/BCd); and Odds Ratios and 95% confidence intervals for tubular kidney dysfunction in relation to bioindicators of Cd exposure and Zn status. Tubular kidney dysfunction: B2M in urine >300 µg/g creatinine. Odds Ratios were computed as described in text. (Adapted with permission from Springer Nature. Chen et al. 2018, *Biol. Trace Elem. Res.* [90]).

	n		Odds Ratio (95% CI)
UCd (µg/g cr)	84	Reference (<3.6)	1
	82	3.6–8.7	1.32 (0.56–3.16)
	81	8.7–16.9	2.83 (1.26–6.36)
	84	≥16.9	4.29 (1.93–9.56)
BCd (µg/L)	83	Reference (<1.85)	1
	83	1.85–7.34	1.46 (0.51–4.14)
	83	7.34–14.89	6.30 (2.51–16.11)
	82	≥14.89	10.42 (4.14–26.21)
Zn/Cd	83	Reference (<100.0)	1
	88	100.0–200.0	0.47 (0.24–0.89)
	81	200.0–600.0	0.16 (0.07–0.36)
	79	≥600.0	0.06 (0.02–0.16)
SZn (mg/L)	111	Reference (<1.14)	1
	171	1.14–1.62	0.66 (0.39–1.14)
	49	≥1.62	0.38 (0.12–1.23)
HZn (mg/g)	42	Reference (<0.12)	1
	48	0.12–0.14	0.57 (0.18–1.83)
	58	≥0.14	0.12 (0.03–0.49)

## Data Availability

Not applicable.
